# Annual acknowledgement of reviewers

**DOI:** 10.1186/1471-2148-14-22

**Published:** 2014-01-29

**Authors:** Christopher Foote

**Affiliations:** 1 BioMed Central, 236 Gray’s Inn Road, London, WC1X 8HB, UK

## Abstract

**Contributing reviewers:**

The editors of BMC Evolutionary Biology would like to thank all our reviewers who have contributed to the journal in Volume 13 (2013).

## 

Amir Abbasi

Pakistan

Jessica Abbott

Sweden

Stephen Abedon

USA

Teresa Adell

Spain

Amir Aharoni

Israel

Tom Aire

Grenada

Fernando Alda

Spain

Samuel Alizon

France

Luke Alphey

UK

Elizabeth Alter

USA

Andrei Alyokhin

USA

Ariel Amadio

Argentina

William Amos

UK

Ramón Anadón

Spain

Nikos Andreakis

Australia

Jose Andres

Canada

Maria Anisimova

Switzerland

Jérémy Anquetin

Switzerland

Nils Anthes

Germany

Kevin Arbuckle

UK

Devin Arbuthnott

Canada

Miguel Arenas

Spain

Tomotsugu Arikawa

Japan

Pim Arntzen

Netherlands

Wolfgang Arthofer

Austria

Tom Artois

Belgium

Marina Ascunce

USA

Raquel Assis

USA

Steven Austad

USA

Ricardo Azevedo

USA

Eric Baack

USA

Simon Babayan

UK

Courtney Babbitt

USA

Charles Bacon

USA

Mounawer Badri

Tunisia

Eva Baermann

UK

Justin Bahl

USA

Evgeniy Balakirev

USA

Francisco Balao

Spain

Guillaume Balavoine

France

Sebastian Baldauf

Germany

Oliver Balmer

Switzerland

David Baltrus

USA

Mukul Bansal

USA

Nigel Barker

South Africa

Tim Barraclough

UK

Craig Barrett

USA

Oguz Baskurt

Turkey

Jake Baum

Australia

Line Bay

Australia

Peter Beerli

USA

Jasminca Behrmann-Godel

Germany

Charles Bell

USA

Michael Bell

USA

Mauricio Bellon

Italy

Alberto Benguria

Spain

Bastian Bentlage

USA

Michael Berenbrink

UK

Camillo Berenos

UK

Fabio Bernini

Italy

Chelsea Berns

USA

Stephanie Bertrand

France

Ricardo Betancur-R.

Colombia

Mireille Betermier

France

Stephen Beverley

USA

Etienne Bezault

USA

Kaspar Bienefeld

Germany

Kuke Bijlsma

Netherlands

Jean-Christophe Billeter

Netherlands

William Black

USA

David Blair

Australia

Christoph Bleidorn

Germany

Roger Blench

UK

Paul Bloor

Colombia

Alexander Bolshoy

Israel

Richard Bomphrey

UK

Simona Boncompagni

Italy

Michael Bonkowski

Germany

Lisa Bono

USA

Alex Bortvin

USA

Kostantinos (Kostas) Bourtzis

Austria

Brian Bowen

USA

Heather Bracken-Grissom

USA

Brenda Bradley

USA

Bart Braeckman

Belgium

Edward Braun

USA

Felix Breden

Canada

Antonio Brehm

Portugal

Patricia Brennan

USA

Amanda Bretman

UK

Henner Brinkmann

Canada

Janice Britton-Davidian

France

Luciano Brocchieri

USA

Michael Brockhurst

UK

Denis Brothers

South Africa

Chris Brown

USA

Richard Brown

UK

Karen Browning

USA

Robb Brumfield

USA

David Bryant

New Zealand

Josef Bryja

Czech Republic

Rob Bryson

USA

Ann Budd

USA

Jim Bull

USA

Francesc Calafell

Spain

Carolina Calviño

Argentina

Kenneth Cameron

USA

Stephen Cameron

Australia

Michael Campbell

USA

Simona Candiani

Italy

Aurore Canoville

South Africa

Andrea Cardini

Italy

Margarida Cardoso Moreira

USA

Salvador Carranza

Spain

Anne-Ruxandra Carvunis

USA

Claudio Casola

USA

Todd Castoe

USA

Isa Cavaco

Sweden

Matteo Cavaliere

Spain

Domitille Chalopin

France

Belinda Chang

Canada

Hubert Charles

France

Mark Chase

UK

Fabrice Chatonnet

France

Lars Chatrou

Netherlands

Raquel Chaves

Portugal

Kaifu Chen

USA

Zhiduan Chen

China

Mark Christie

USA

Dong Chu

China

Alice Cibois

Switzerland

Alexandra Cieslak

Spain

Jihong Liu Clarke

Norway

Christopher Cleal

UK

Sonya Clegg

Australia

Martin Coetzee

South Africa

Lark Coffey

USA

Julie Collet

UK

Helene Collin

UK

Allen Collins

USA

Michael Collyer

USA

Ian Colquhoun

Canada

Gavin Conant

USA

Fabien Condamine

France

Jan Conn

USA

James Cook

UK

Alex Cordoba-Aguilar

Mexico

Miguel Corona

USA

Andrea Cosacov

Argentina

Maria Costantini

Italy

Joel Cracraft

USA

Keith Crandall

USA

Peter Crane

USA

Chiquito Crasto

USA

Bernard Crespi

Canada

Trey Crisco

USA

Glynis Cron

South Africa

Fulvio Cruciani

Italy

Tami Cruickshank

USA

Miklos Csuros

Canada

Liwang Cui

USA

Ana Paula Cutrera

Argentina

Joel Dacks

Canada

Tal Dagan

Germany

Ann Daly

UK

Alejandro D'Anatro

Uruguay

Alistair Darby

UK

Liliana Davalos

USA

Guillaume Daver

France

William Davidson

Canada

Julia Day

UK

Kenneth De Baets

Switzerland

Olivier De Clerck

Belgium

Peter de Knijff

Netherlands

A. P. Jason De Koning

Canada

Jeremy Debarry

USA

Ellen Decaestecker

Belgium

Sandie Degnan

Australia

Massimo Delfino

Italy

John Dennehy

USA

Annette Denzinger

Germany

Miroslava Derenko

Russia

Stephane Derocles

UK

Patrizia d'Ettorre

France

Andrew Dewoody

USA

Richard Di Giulio

USA

Francisco Dionisio

Portugal

Stephen Dobson

USA

Julian Dodson

Canada

Cristina Domingo

Spain

Helen Donoghue

UK

Russell Doolittle

USA

Alex Dornburg

USA

Emmanuel Douzery

France

Mark Dowton

Australia

Lee Drickamer

USA

William Driscoll

USA

Lei Du

USA

Meghan Duffy

USA

Siobain Duffy

USA

Hannah Dugdale

UK

David Duneau

USA

Julie Dunning Hotopp

USA

Stéphane Dupas

Ecuador

Carlo Duso

Italy

Paul Eady

UK

Ingo Ebersberger

Germany

Pim Edelaar

Spain

Dominic Edward

UK

Torbjorn Ekrem

Norway

Mark Eldridge

Australia

Allan Ellis

South Africa

Brian Ellis

Canada

Eyal Elyashiv

Israel

Richard Emes

UK

John A Endler

Australia

Agustin Estrada-Pena

Spain

Rampal Etienne

Netherlands

Alistair Evans

Australia

Melissa Evans

Canada

Arnaud Faille

Germany

Marie-Julie Favé

Canada

Judith Fehrer

Czech Republic

Lars Fehre-Schmitz

USA

Brock Fenton

Canada

Juan Fernandez-M.

France

Philine G. D. Feulner

Germany

Pedro Fiaschi

Brazil

Renee Firman

Australia

Omar Fiz-Palacios

Sweden

Diego Fontaneto

Italy

Gianluigi Forloni

Italy

Mattias Forshage

Sweden

Brett Forshey

USA

Richard Fortey

UK

Greg Fournier

USA

Lucia Franchini

Argentina

Bonnie Fraser

Germany

Ceridwen Fraser

Belgium

Loreta Freitas

Brazil

Robert Friedman

USA

Markus Friedrich

USA

Peter Fritsch

USA

Matthew Fujita

USA

Peter Funch

Denmark

Toni Gabaldon

Spain

Holly Gaff

USA

Pierre-Alexandre Gagnaire

Canada

Oliver Gailing

USA

Philippe Gambette

France

Song Gang

China

Fernando Garcia-Arenal

Spain

Francisco Garcia-Gonzalez

Australia

Jose-Luis García-Marín

Spain

David Garfield

Germany

John Gatesy

USA

Eric Gaucher

USA

Philippe Gayral

France

Xuejun Ge

China

Gillian Gibb

New Zealand

Raymond Gill

USA

Tatiana Giraud

France

Gonzalo Giribet

USA

Thomas Givnish

USA

Frank Glaw

Germany

Greg Gloor

Canada

Matthew Goddard

New Zealand

Alfred Goldberg

USA

G Brian Golding

Canada

Sharyn Goldstien

New Zealand

Elena Gomez

USA

Xun Gong

China

Benjamin Good

USA

Uri Gophna

Israel

Alexander Gorbalenya

Netherlands

Isabel Gordo

Portugal

Marc Gottschling

Germany

Fred Gould

USA

Per Einar Granum

Norway

Emma Greig

USA

John M. Grindeland

Norway

Konstantin Gunbin

Russia

An-Yuan Guo

China

Vijai Kumar Gupta

Ireland

Martin Haase

Germany

Pascal Hablützel

Belgium

Annelies Haegeman

Belgium

Matthew Hahn

USA

Frank Hailer

USA

Vladimir Hampl

Czech Republic

James Hamrick

USA

Tyler Harp

USA

D. James Harris

Portugal

Alan Harvey

USA

Michael Harvey

USA

John Hash

USA

Mark Hauber

USA

Lorenz Hauser

USA

Ronald Hay

UK

David Haymer

USA

Alexander Hayward

Sweden

Tracy Heath

USA

Chris Heesy

USA

Andrew Heidel

Germany

Heikki Helanterä

Finland

Johannes Helder

Netherlands

Andrew Hendry

Canada

Brenna Henn

USA

Gabor Herczeg

Hungary

Russell Hermansen

USA

Anthony Herrel

France

Israel Hershkovitz

Israel

Katja Heubel

Germany

Myriam Heuertz

Belgium

Michael Hiller

Germany

Jason Hilton

UK

Axel Hochkirch

Germany

Bert Hoeksema

Netherlands

Herbert Hoi

Austria

Mark Holder

USA

Michael Holick

USA

Barbara Holland

New Zealand

Peter W.H. Holland

UK

Luke Holman

Australia

Ian Holmes

USA

Rodney Honeycutt

USA

David Hoogewijs

Switzerland

Eberhard Horn

Germany

Conrad Hoskin

Australia

Thomas Hossie

Canada

Andreas Houben

Germany

Massoud Houshmand

Iran

Howard Howland

USA

Mark Hughes

UK

Nigel Hughes

USA

Ann Kathrin Huylmans

Germany

Shih-Ying Hwang

Taiwan

Javier Igea

UK

Boris Igic

USA

Chris Illingworth

UK

Simone Immler

Sweden

Wendy Ingram

USA

Manuel Irimia

Canada

Iker Irisarri

Germany

David Irwin

Canada

Nina Jablonski

USA

Jan Janecka

USA

Axel Janke

Germany

Sharon Jansa

USA

Daniel Jeffares

UK

Ronald Jenner

UK

Frank Johansson

Sweden

Steig Johnson

Canada

Vida Jojic Sipetic

Serbia

Adam Jones

USA

Gerrit Joop

Germany

Leo Joseph

Australia

Carlos Juan

Spain

Brent Kaiser

Australia

Antigoni Kaliontzopoulou

Portugal

Olga K. Kamneva

USA

Rosa Karlic

Croatia

Vaishali Katju

USA

Akito Yuji Kawahara

USA

Shinpei Kawaoka

Japan

Darcy Kelley

USA

Morgan Kelly

USA

Martyn Kennedy

New Zealand

Hossein Khiabanian

USA

Dongsup Kim

South Korea

Wook Kim

USA

Evan Kingsley

USA

Roland Kirschner

Taiwan

Eva Kisdi

Finland

Hirohisa Kishino

Japan

Oddmund Kleven

Norway

John Klicka

USA

Jennifer Knies

USA

Matthew Knope

USA

Ichizo Kobayashi

Japan

Ullasa Kodandaramaiah

India

Astrid Kodric-Brown

USA

Mathias Koelliker

Switzerland

Fyodor Kondrashov

Spain

Ales Kovarik

Czech Republic

Simon Krattinger

Switzerland

Anton Kratz

Japan

Daniel Krupp

Canada

Shigehiro Kuraku

Japan

Maurizio Labbate

Australia

Simon Lailvaux

USA

Kathrin Lampert

Germany

Giddy Landan

USA

Michael Lang

France

Bernd Markus Lange

USA

Gordon Langsley

France

Dan Larhammar

Sweden

Amparo Latorre

Spain

Sacha Laurent

Switzerland

Simon Laurin-Lemay

Canada

Brian Lazzaro

USA

Adam Leache

USA

Christophe Lebigre

Belgium

Walter Lechner

Austria

David Legg

UK

Richard Lehtinen

USA

Jussi Lehtonen

Australia

Tuomas Leinonen

Finland

Andrew Leitch

UK

Tobias Lenz

Germany

Hannes Lerp

Germany

Enrique P. Lessa

Uruguay

Anthony Levasseur

France

Michael Levin

USA

Andre Levy

Portugal

Gwilym Lewis

UK

Mary Lewis

UK

Zenobia Lewus

UK

Yuhe Liang

USA

Morten Lillemo

Norway

Zoe Lindo

Canada

Jianquan Liu

China

Jin-Xian Liu

China

Tao Liu

China

Zhong-Jian Liu

China

Andrew Lloyd

Ireland

Peter Lockhart

New Zealand

Tristan Long

Canada

Xavier Lopez

Spain

Carlos Lopez-Vaamonde

France

Elgion Loreto

Brazil

Stephen Lougheed

Canada

Edward Louis

USA

Anice Lowen

USA

Xiongbin Lu

USA

Kay Lucek

Switzerland

Andrea Luchetti

Italy

Patricia Lu-Irving

USA

Donata Luiselli

Italy

Julius Lukes

Czech Republic

Stefan Lupold

USA

Jiri Macas

Czech Republic

John Mackrill

Ireland

Thomas Mailund

Denmark

Izabela Makalowska

Poland

Ray Malfavon-Borja

USA

Ludovic Mallet

France

Ferruccio Maltagliati

Italy

Steven Manchester

USA

Marina Marcet-Houben

Spain

Frantisek Marec

Czech Republic

David Mark Welch

USA

William Martin

Germany

Julien Martinez

UK

Paulino Martínez

Spain

Neus Martínez Abadías

Spain

Alino Martinez-Marcos

Spain

Inigo Martinez-Solano

Spain

Joanna Masel

USA

Daniel Masiga

Kenya

Leila Masri

Austria

Steven Massey

Puerto Rico

Ricardo Masuelli

Argentina

Sarah Mathews

USA

Atsushi Matsui

Japan

Masafumi Matsui

Japan

Conrad Matthee

South Africa

Scott McCairns

Finland

John McCormack

USA

Megan McCusker

Canada

John McCutcheon

USA

Stuart McDaniel

USA

Allan McDevitt

Canada

Tami McDonald

USA

James McInerney

Ireland

Jonathan Mee

Canada

Patrick Meirmans

Netherlands

Richard Meisel

USA

Tamra Mendelson

USA

Stefan Merker

Germany

Heidi Meudt

New Zealand

Joel Meunier

Germany

Johan Michaux

Belgium

Andy Michel

USA

Michel Milinkovitch

Switzerland

Andrew Miller

USA

Alessandro Minelli

Italy

Aurélien Miralles

Germany

Philipp Mitteroecker

Austria

Anders Pape Moller

France

Michael Möller

UK

Daniel Money

USA

Cara Monroe

USA

Valeria Montano

Austria

Leandro Monteiro

Brazil

Michael Moody

USA

Andrew D. Moore

Germany

Patricia Moore

USA

Levi Morran

USA

David Morrison

Sweden

Edward Morrow

UK

Alan Moses

Canada

Masaharu Motokawa

Japan

Adnan Moussalli

Australia

Timothy Mousseau

USA

Kai Mueller

Germany

Rachel Mueller

USA

Sean Mullen

USA

Joaquin Munoz

Spain

Violeta Munoz-Fuentes

Spain

Maria Anice Mureb Sallum

Brazil

Ben Murrell

USA

Delphine Muths

France

Sean Myles

Canada

Benoit Nabholz

France

Kiyotaka Nagaki

Japan

Cleusa Nagamachi

Brazil

Valakunja Nagaraja

India

Haruki Nakamura

Japan

Luay Nakhleh

USA

Kiyoshi Naruse

Japan

Santiago Nava

Argentina

Nicolas Navarro

France

Thomas Near

USA

Linda Neaves

UK

Richard Neher

Germany

Volker Nehring

Germany

Andre Nel

France

Martha Nelson

USA

Martha Nelson-Flower

South Africa

Scott Nichols

USA

Oliver Niehuis

Germany

Matthew Niemiller

USA

Yoshihito Niimura

Japan

Fred Nijhout

USA

Naruo Nikoh

Japan

Deng-Ke Niu

China

Fabio Nogueira

Brazil

Suzuki Noriyuki

Japan

Benjamin Normark

USA

Tetyana Nosenko

Germany

Carsten Nowak

Germany

Dmitry Nurminsky

USA

Darren Obbard

UK

Benedikt Obermayer

USA

John Obrycki

USA

Anders Odeen

Sweden

Jan Oettler

Germany

Oivind Oines

Norway

Dario Ojeda Alayon

Canada

Matthew Oliver

UK

Anna Olivieri

Italy

Juan Opazo

Chile

Claudia Patricia Ornelas-Garcia

Mexico

Megan Osborne

USA

Benjamin Oswald

USA

Charles Oxnard

Austria

Marie Pagès

France

Athma Pai

USA

Csaba Pal

Hungary

Maria Pala

UK

Francisco Panzera

Uruguay

Anthony Papenfuss

Australia

Theodore Papenfuss

USA

Kevin Parsons

UK

Adrian Paterson

New Zealand

Bret Pearson

Canada

David J Penman

UK

David Penny

New Zealand

Ugo Alessandro Perego

USA

S. Ivan Perez

Argentina

Adrien Perrard

France

Jennifer Perry

UK

Ralph Peters

Germany

Eric Petit

France

Andrea Pilastro

Italy

Catarina Pinho

Portugal

Joao Pinto

Portugal

Paolo Piras

Italy

Michael David Pirie

South Africa

Davide Pisani

UK

Martin Plath

Germany

Helmut Plattner

Germany

Lars Podsiadlowski

Germany

Hendrik Poinar

Canada

Andrew Pomiankowski

UK

Julia Poncela-Casasnovas

USA

Megan Porter

USA

Michelle Power

Australia

Simon Powers

Switzerland

Brian Preston

UK

Julien Prunier

Canada

David Punzalan

Canada

Mike Purdy

USA

Mikael Puurtinen

Finland

Yingxiong Qiu

China

Yin-Long Qiu

USA

Liang-Hu Qu

China

Marcel Quint

Germany

Joost Raeymaekers

Switzerland

Jennifer Raff

USA

Satyawada Rama Rao

India

Steven Ramm

Germany

Didier Raoult

France

Mark Rausher

USA

Timothy Read

USA

Brian Rector

USA

David L. Remington

USA

Sabrina Renaud

France

Justin Rheubert

USA

Ângela Ribeiro

Portugal

Ignacio Ribera

Spain

David Richardson

UK

Stefan Richter

Germany

Robert Ricklefs

USA

Markus Riegler

Australia

Monique Rijnkels

USA

Peter Ritchie

New Zealand

Dale Roberts

Australia

Francisco Rocha

Spain

Sara Rocha

Portugal

Josephine Rodriguez

USA

Francisco Rodriguez-Valera

Spain

Kim Roelants

Belgium

Rebekah Rogers

USA

Igor Rogozin

USA

Maria Ines Roldan

Spain

Isabel Roldán-Ruiz

Belgium

Campbell Rolian

Canada

Pedro Romano

Brazil

Jonathan Romiguier

France

Eliora Ron

Israel

Marilyn Roossinck

USA

Cristina Roquet

France

Laura Rose

Germany

Rebeca Rosengaus

USA

Balázs Rosivall

Hungary

Marie-Noëlle Rosso

France

Omar Rota-Stabelli

Italy

Olivia Roth

Germany

Stefan Rothenburg

USA

Beatrice Roure

Canada

Julien Roux

USA

Kevin Rowe

Australia

Scott Roy

USA

Julio Rozas

Spain

Nimrod Rubinstein

Israel

Andreas Rudh

Sweden

Carlos Ruiz

Ecuador

Iñaki Ruiz-Trillo

Spain

Howard Rundle

Canada

Anna Runemark

Sweden

Francois Russet

France

Danilo Russo

Italy

Paul Rymer

Australia

Kalle Rytkonen

USA

Urmas Saarma

Estonia

Beatriz Sabater-Muñoz

Spain

Antonio Salas

Spain

Daniele Salvi

Portugal

Diego San Mauro

Spain

Thomas Sanger

USA

Isabel Sanmartin

Spain

Jordan Satler

USA

Herve Sauquet

France

Laura Scheinfeldt

USA

Christian Schlötterer

Austria

Thomas Schmitt

Germany

Juergen Schmitz

Germany

Lars Schmitz

USA

James Schnable

USA

Korbinian Schneeberger

Germany

Gerald M. Schneeweiss

Austria

Conrad Schoch

South Africa

Astrid Schoen

Germany

Sean Schoville

USA

Patricia Schulte

Canada

Tanja Schwander

Switzerland

Marilyn Scott

Canada

Jeremy Searle

USA

Ole Seehausen

Switzerland

Laure Segurel

USA

Jae Young Seong

South Korea

Jeanne Serb

USA

Joao Setubal

Brazil

Jan Sevcik

Czech Republic

Aaron Shafer

Sweden

Tamim Shaikh

USA

Craig Sherman

Australia

Emma Sherratt

USA

Nancy Sherwood

Canada

Seema Sheth

USA

Norito Shibata

Japan

Masaki Shimamura

Japan

L. David Sibley

USA

Julia Sigwart

UK

Derek Sikes

USA

Johannes Sikorski

Germany

Michael Simone-Finstrom

USA

Tatum Simonson

USA

Anna Skoracka

Poland

Konstantin Skryabin

Russia

Nina Sletvold

Sweden

Daniel Sloan

USA

Jason Slot

USA

James Smith

USA

Joe Smith

USA

Leo Smith

USA

Paul Smith

USA

Terry Smith

UK

Marinus Smulders

Netherlands

Darren Soanes

UK

Nick Sokol

USA

Simone Sommer

Germany

Montuire Sophie

France

Teiji Sota

Japan

Victor G. Springer

USA

Roscoe Stanyon

Italy

James Starrett

USA

Juliana Sterli

Argentina

Caitlin Stern

USA

Michael Stich

USA

Mathias Stiller

Germany

Anne Stone

USA

Astrid V. Stronen

Poland

Gary Stuart

USA

Kenta Sumiyama

Japan

Zuzana Swigonova

USA

Yuma Takahashi

Japan

Koji Takayama

Austria

Nadia Talent

Canada

Kristiina Tambets

Estonia

Mehmet Tan

Turkey

Qiongying Tang

China

Oznur Tastan

Turkey

Amber Teacher

UK

Leho Tedersoo

Estonia

Ilya Temkin

USA

Kristin Tessmar-Raible

Austria

Francoise Thibaud-Nissen

USA

Michael Thon

Spain

Michaela Thoß

Austria

Stephen Tilley

USA

Elisabeth Tillier

Canada

Michael Tobler

USA

Sergio Tofanelli

Italy

Ave Tooming-Klunderud

Norway

Luis M. Torres-Vila

Spain

Jeffrey Townsend

USA

Ted Townsend

USA

Costas Triantaphyllidis

Greece

Vladimir Trifonov

Russia

Wen-Chieh Tsai

Taiwan

Neil Tsutsui

USA

Paul Turner

USA

Alex Twyford

USA

Jeremy Ullmann

Australia

Peter Underhill

USA

Turgay Unver

Turkey

Pedro Vale

France

Yves Van de Peer

Belgium

Ron Van Den Bussche

USA

Arie van der Meijden

Portugal

Johanna H.A. van Konijnenburg-van Cittert

Netherlands

Cock van Oosterhout

UK

Alain Vanderpoorten

Belgium

Maarten Vanhove

Belgium

Krisztina Varga

USA

Daniel Vasco

USA

Raquel Vasconcelos

Portugal

Hubert Vaudry

France

Manuel Vera

Spain

Philippe Vernier

France

Diego H. Verzi

Argentina

Marcelo Ricardo Vicari

Brazil

Tom Vogwill

UK

Björn Marcus von Reumont

Germany

Hiroshi Wada

Japan

Philipp Wagner

USA

Niklas Wahlberg

Finland

David Wake

USA

Ross Frederick Waller

Australia

Graham Wallis

New Zealand

Xiu-Jie Wang

China

Andrew Wargo

USA

Yasuyuki Watano

Japan

Ward Watt

USA

Fuwen Wei

China

Alexander Weigand

Germany

Ariel Weinberger

USA

Daniel Weinreich

USA

James B Whitfield

USA

Danielle Whittaker

USA

Claude Wicker-Thomas

France

Martin Wiemers

Germany

Stuart Wigby

UK

Claus Wilke

USA

Endre Willassen

Norway

Kristie Willett

USA

Alex C. C. Wilson

USA

Christopher Wilson

UK

Megan Wilson

New Zealand

Benedicte Wirth

France

Justyna Wolinska

Germany

Alex Wong

Canada

Alexandra Worden

USA

Shuangxiu Wu

China

Jenny Xiang

USA

Nabila Yahiaoui

France

Xiao-Jun Yang

China

Krassimir Yankulov

Canada

Chen-Hsiang Yeang

Taiwan

Anne Yoder

USA

Kazunori Yoshizawa

Japan

Li Yu

China

Kei Yura

Japan

Christina Zakas

USA

Luis Zaman

USA

Peng Zhang

China

Shiliang Zhou

China

Kirk Zigler

USA

Robert Zink

USA

Christian Zmasek

USA

Mark Zwart

Spain

